# Self-medication with antibiotics during the COVID-19 pandemic: A cross-sectional study among adults in Tema, Ghana

**DOI:** 10.1371/journal.pone.0305602

**Published:** 2024-06-25

**Authors:** Henry Kwadwo Hackman, Lawrence Annison, Reuben Essel Arhin, George Osei Adjei, Phyllis Otu, Emele Arthur-Hayford, Sharon Annison, Bernard Bortei Borteih

**Affiliations:** 1 Department of Medical Laboratory Technology, Faculty of Applied Sciences, Accra Technical University, Accra, Ghana; 2 Department of Science Laboratory Technology, Faculty of Applied Sciences, Accra Technical University, Accra, Ghana; 3 Department of Epidemiology and Disease Control, School of Public Health, University of Ghana, Accra, Ghana; 4 Department of Animal Research Institute, Council for Scientific and Industrial Research (CSIR), Accra, Ghana; Wollega University, ETHIOPIA

## Abstract

**Background:**

Antibiotic self-medication is one of the common causes of antibiotic resistance of bacterial organisms. The COVID-19 pandemic introduced a new paradigm shift and significantly influenced healthcare behaviors, including an increase in antibiotic self-medication, which contributes to antibiotic resistance. This study was aimed at determining the prevalence of antibiotic self-medication and the possible associated factors during the peak of the COVID-19 pandemic among adult residents of Tema in Ghana from April to July 2021.

**Methods:**

Using a cross-sectional design, 400 adults were randomly selected and surveyed using a researcher-assisted questionnaire. Data were analyzed with IBM® SPSS® Statistics Version 22.0, considering associations significant at a 95% confidence interval (p < 0.05).

**Results:**

Of the 400 respondents, (76%) 304 had practiced antibiotic self-medication within the previous 12 months during the COVID-19 pandemic. Significant factors associated with antibiotic self-medication included gender, age, marital status, education, occupation, and National Health Insurance Scheme subscription. Convenience and avoiding long hospital queues were primary non-medical reasons for antibiotic self-medication, while previous successful experience, easy access to antibiotics, treating symptoms, prophylaxis, and fear of hospital infection were the medical reasons for antibiotic self-medication. Commonly self-administered antibiotics were azithromycin (34%), amoxicillin/clavulanic acid (22%), and metronidazole (16%) for perceived respiratory tract and gastrointestinal tract infections.

**Conclusions:**

The high prevalence of antibiotic self-medication observed during the COVID-19 pandemic underscores the need for enhanced public education and stricter enforcement of regulations governing antibiotic sales. The non-medical and medical factors of convenience, avoiding long hospital queues, previous successful experience, easy access to antibiotics, treating symptoms, prophylaxis, and fear of hospital infection which motivated antibiotic self-medication practices require the implementation of antimicrobial stewardship interventions.

## Introduction

Self-medication is the practice of a personal usage of medications to treat diseases without prescription from a qualified clinician. This practice is prevalent across several third world countries and Ghana is no exception. The medication mostly includes commonly used antimicrobials used to treat communicable diseases such as viral, bacterial, and parasitic infections. Antibiotic self-medication is one of the common causes of antibiotic resistance of bacterial organisms [1].

Antibiotics are mostly prescriptive drugs used to treat bacterial infections. Antibiotics are one of the most used drugs in developing countries due to the high levels of infectious diseases [2]. Studies have indicated an increase in the multi-drug resistance patterns among bacterial organisms in the population. The prevalence of multi-drug resistant bacterial organisms is due to the abuse and misuse of antibiotics in humans and animals. The resistant bacterial stains often lead to increase morbidity and mortality rates among patients, high cost of quality healthcare and loss of man hours [3]. The socio-economic outcome of high multi-drug resistances due to antibiotic self-medication is a public health emergency that demands deliberate attention by policy makers and implementers.

Several factors have been enumerated as the associated factors for the high prevalence of antibiotic self-medication. These include past successful experience, easy access to antibiotics and high prevalence of infectious diseases such as diarrhoae, respiratory infections [4]. The COVID-19 pandemic which was announced in Ghana in March 2020 also introduced a new paradigm shift in the associated factors for antibiotic self-medication. The COVID-19 pandemic is a critical source of antibiotic self-medication and antimicrobial resistance since a significant number of SARS-CoV-2-exposed persons may not seek medical attention in the hospital due to several reasons [5].

Empirical antibacterial therapy has been widely reported since the inception of COVID-19 pandemic. Previous studies in Wuhan indicated that over 90% of COVID-9 patients who were admitted into health facilities were given antibiotics with no evidence of bacterial infection [6]. Studies by Langford *et al*., established that 71.3% of COVID-19 patients were prescribed antibiotics by recognized hospitals [7].

The empiric antibacterial treatment of COVID-19 patients in health facilities is a major risk for increasing antibiotic resistance in the population [8]. These antibacterial therapy for COVID-19 patients were initiated with little or no evidence of bacterial co-infection [9]. Among the few coinfections, the prevalent respiratory infections which were isolated among COVID-19 patients were *Staphylococcus aureus*, *Pseudomonas* species, and *Klebsiella* species, and the most common blood culture isolates were *Staphylococcus* aureus, *Staphylococcus epidermidis*, and *Streptococcus* species [10]. Nori *et al* reported that despite the low (3.6%) incidence of confirmed coinfection, 98% of hospitalized COVID-19 patients were treated with antibiotics [10]. The misuse of antibiotics during the COVID-19 pandemic is a recipe for public health emergency [11].

To the best of our knowledge, there is no published information of the ASM among residents in Tema. This cross-sectional study was aimed at determining the occurrence of antibiotic self-medication and the possible associated factors during the peak of the COVID-19 pandemic among adult residents of Tema in Ghana.

## Materials and methods

### Study setting

The study was undertaken among residents of Tema in the Greater Accra Region of Ghana. Tema, which is the capital city of the Tema Metropolitan Assembly, is a harbour and industrial city. According to the 2021 Population and Housing Census, Tema has 175,688 households and the total population of Tema is 177,924 with 87,529 males and 90,395 females [12]. The population is distributed in an area of 565 km^2^ (218 sq mi) [12]. The Greenwich Meridian (00 Longitude) passes directly through Tema [12].

### Study design

The study was a cross-sectional study conducted among adult residents of Tema from April to July 2021 to determine the occurrence and associated factors of antibiotic self-medication during the COVID-19 pandemic.

### Study population, sampling technique, questionnaire, and data collection

Residents of Tema who were 18 years and above and have previous self-use of antibiotics in the past 12 months were included in the survey.

A random sampling technique was employed to recruit 400 respondents and a researcher-assisted questionnaire was used to obtain quantitative responses from the participants [4, 13]. Randomly selected residents for not less than three months were included in the population survey. A questionnaire was developed consisting of both closed and open-ended questions and was in two parts. The socio-demographic characteristic (age, gender, marital status, education, occupation, NHIS subscription) constituted the first part and the second part was data on previous antibiotic self-medication in the past 12 months (knowledge, attitude, course of treatment, condition of health that necessitated antibiotic self-medication, source of antibiotics, reasons for antibiotic self-medication, antibiotics and source of information). The respondents who agreed to participate in the study were interviewed for about 20–25 minutes in their respective residences. Antibiotics commonly used in Tema were used to help the respondents to recall the names of previously used antibiotics.

### Sample size determination

The sample size (n) was calculated using the Sample Size for One Sample, Dichotomous Outcome where n is given as $n=\frac{p_{0}q_{0}{\left\{Z_{1-\frac{\alpha }{2}}+Z_{1-\beta }\sqrt{\frac{p_{1}q_{1}}{p_{0}q_{0}}}\right\}}^{2}}{{\left(p_{1}-p_{0}\right)}^{2}}$

Where p_0_ = proportion (incidence) of population

p_1_ = proportion (incidence) of study group

n = sample size for study group

α = probability of type I error (usually 0.05)

β = probability of type II error (usually 0.2)

z = critical Z value for a given α or β

Given the p_0_ = proportion (incidence) of population = 70% [13], Estimated proportion (incidence) of study group p_1_ = 76%, α = 0.05 and β = 0.15. Considering a Power of 85%, final required sample size was estimated at 499. The study was able to recruit 400 (80%) out of the proposed sample size. The reason was the availability of respondents that satisfy the inclusion criteria.

### Survey variables

The independent variables in the study were socio-demographic characteristics such as age, gender, marital status, education, occupation, NHIS subscription, previous antibiotic self-medication in the past 12 months, knowledge, attitude, course of treatment, condition of health that necessitated antibiotic self-medication, source of antibiotics, reasons for antibiotic self-medication, name of antibiotics and source of information. The main outcome variable was antibiotic self-medication among residents of Tema in the Greater Accra Region, Ghana during the COVID-19 pandemic.

### Data processing and statistical analyses

The data generated was analysed using IBM^®^ SPSS^®^ Statistics Version 22.0. The data was presented in the form of frequencies and percentages using univariate analysis on the socio-demographic characteristics of respondents. Bivariate comparisons were used to determine the association between ASM and independent variables, associated factors of ASM and prevalence ratios (PRs) using chi square test. Associations were considered significant at 95% confidence interval with p value set at *<*0.05. The independent variables that were significantly associated with ASM were employed in a multivariate analysis using logistic regression to establish the effect of the associated factors on ASM.

### Ethical clearance

The protocol used for data collection was approved by the Ethical Review Committee of Accra Technical University with protocol identification number ATU/MLT/ET/003/2020-2021. The objectives of the study, risks, benefits, right to refuse and confidentiality were explained to the respondents prior to obtaining written informed consent to voluntarily participate in the survey. The identity and information on the respondents were not disclosed. The authors do not have access to information that could identify individual participants during or after data collection.

## Results

### Socio-demographic characteristics of respondents

Most of the respondents, 248 (62%) out of the 400 were females. The largest age group 114 (28.5%) were between the ages of 25 and 35. Two hundred and fifty (250) which is 62.5% of respondents were married. Most respondents, 158 (39.5%) had completed at least secondary school education, while 132 (33%) were mostly employed in the public sector. Regarding National Health Insurance Scheme (NHIS) subscription, 250 (62.5%) had subscribed while 150 (37.5%) were non-subscribers as shown in (Table 1).

**Table 1:** Socio-demographic characteristics of respondents (n = 400).

Variable	Category	Count	Frequency
**Gender**	**Male**	152	38.0%
	**Female**	248	62.0%
	**Total**	400	100.0%
**Age**	18–24	86	21.5%
	25–35	114	28.5%
	36–45	95	23.8%
	46–55	64	16.0%
	56–65	31	7.8%
	> 65	10	2.5%
**Marital Status**	Single	135	33.8%
	Married	250	62.5%
	Divorced	15	3.8%
**Education**	Illiterate	32	8.0%
	Basic	150	37.5%
	Secondary	158	39.5%
	Tertiary	60	15.0%
**Occupation**	Public Sector	132	33.0%
	Private Sector	105	26.3%
	Self Employed	98	24.5%
	Unemployed	40	10.0%
	Student	25	6.3%
**NHIS Subscription**	No	150	37.5%
	Yes	250	62.5%

### Prevalence of antibiotic self-medication and association with socio-demographic characteristics

Of the 400 respondents, 304 (76%) had practiced antibiotic self-medication within the previous 12 months during the COVID-19 pandemic. There were higher ASM among males and singles than females and married residents respectively. The chi-square analysis showed that the socio-demographic characteristics of participants that significantly correlated with ASM were gender, age, marital status, education, occupation and NHIS subscription (p<0.05) as indicated in Table 2.

**Table 2:** Prevalence of Antibiotic Self-Medication (ASM) and association with socio-demographic characteristics (n = 400).

		Prevalence of Antibiotic Self Medication (%)		Chi Square Test		
Variable	Category	Practice ASM	Does not practice ASM	Chi-square	df	p-value
**Gender**	Male	125 (82.2)	27 (17.8)	5.2	1	0.022
	Female	179 (72.2)	69 (27.8)			
	Total	304 (76)	96 (24)			
**Age**	18–24	84 (97.7)	2 (2.3)	51.3	5	0.001
	25–35	86 (75.4)	28 (24.6)			
	36–45	63 (66.3)	32 (33.7)			
	46–55	51 (79.7)	13 (20.3)			
	56–65	12 (38.7)	19 (61.3)			
	> 65	8 (80)	2 (20)			
**Marital Status**	Single	118 (87.4)	17 (12.6)	14.6	2	0.001
	Married	175 (70)	75 (30)			
	Divorced	11 (73.3)	4 (26.7)			
	Illiterate	25 (78.1)	7 (21.9)			
**Education**	Basic	123 (82)	27 (18)	9.8	3	0.020
	Secondary	119 (75.3)	39 (24.7)			
	Tertiary	37 (61.7)	23 (38.3)			
**Occupation**	Public Sector	84 (63.6)	48 (36.4)	19.9	4	0.001
	Private Sector	90 (85.7)	15 (14.3)			
	Self Employed	82 (83.7)	16 (16.3)			
	Unemployed	29 (72.5)	11 (27.5)			
	Student	19 (76)	6 (24)			
**NHIS**	No	76 (50.7)	74 (49.3)	84.4	1	0.001
	Yes	228 (91.2)	22 (8.8)			

### Relationship between ASM and socio-demographic characteristics

The findings revealed varying prevalence ratio (PR) values along with their associated 95% Confidence Intervals (CIs) for ASM practice within specific socio-demographic cohorts (Table 3). Within the cohort of individuals practicing ASM, those below 25 years showed a significantly higher PR of 1.394 (95% CI: 1.249–1.555). Within the cohort of ASM practitioners, the married respondents exhibited a PR of 1.229 (95% CI: 1.098–1.375), indicating a higher likelihood of self-medicating with antibiotics. The results further showed that within the cohort of ASM practitioners, illiterate individuals displayed a slightly elevated PR of 1.03 (95% CI: 0.915–1.161). Employed individuals showed a slightly higher PR of 1.035 (95% CI: 0.932–1.15), indicating a slightly increased likelihood of ASM practice within this occupational subgroup. Surprisingly, within the cohort of ASM practitioners, those not enrolled in the National Health Insurance Scheme (NHIS) exhibited a lower PR of 0.556 (95% CI: 0.498–0.622) as indicated in Table 3.

**Table 3:** Prevalence ratios (PRs) of ASM practice within socio-demographic cohorts (n = 400).

			95% CI	
Socio-Demographic Variables	Risk Estimate	Value	Lower	Upper
**Age**	Odds Ratio for Age (Below 25 years / ≥ 25 years)	0.056	0.013	0.231
	For cohort Prevalence of Antibiotic Self Medication = Does not practice ASM	0.078	0.02	0.309
	For cohort Prevalence of Antibiotic Self Medication = Practice ASM	1.394	1.288	1.509
**Marital Status**	Odds Ratio for Marital Status (Not Married / Married)	0.38	0.223	0.648
	For cohort Prevalence of Antibiotic Self Medication = Does not practice ASM	0.467	0.301	0.724
	For cohort Prevalence of Antibiotic Self Medication = Practice ASM	1.229	1.108	1.363
**Educational Status**	Odds Ratio for Education (Illiterate / Literate)	0.878	0.367	2.098
	For cohort Prevalence of Antibiotic Self Medication = Does not practice ASM	0.904	0.459	1.784
	For cohort Prevalence of Antibiotic Self Medication = Practice ASM	1.03	0.85	1.249
**Occupation**	Odds Ratio for Occupation (Employed / Unemployed)	0.871	0.474	1.6
	For cohort Prevalence of Antibiotic Self Medication = Does not practice ASM	0.902	0.574	1.416
	For cohort Prevalence of Antibiotic Self Medication = Practice ASM	1.035	0.885	1.21
**NHIS**	Odds Ratio for NHIS (No / Yes)	10.091	5.868	17.353
	For cohort Prevalence of Antibiotic Self Medication = Does not practice ASM	5.606	3.644	8.624
	For cohort Prevalence of Antibiotic Self Medication = Practice ASM	0.556	0.472	0.654
	**N of Valid Cases**	**400**		

### Reasons for antibiotic self-medication

**Fig 1:**
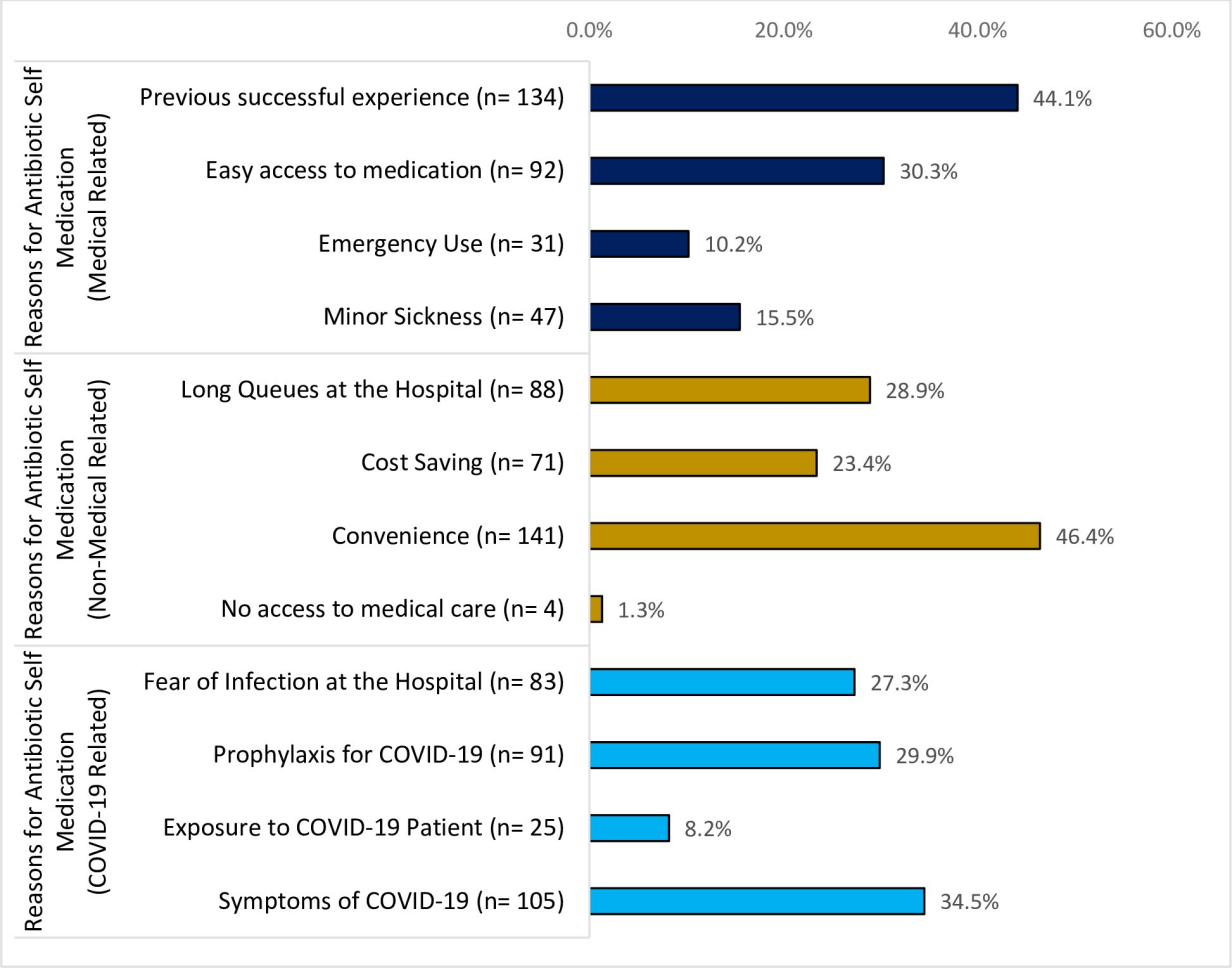
Reasons for antibiotic self-medication (n = 304).

The major medical reasons for ASM were previous successful experience [134 (44.1%)], and easy access to medication [92 (30.3%)]. The major non-medical related reasons for ASM were convenience [141 (46.4%)] and long queues at the hospital [88 (28.9%)]. The major COVID-19 related reasons for ASM included symptoms of COVID-19, [105 (34.5%)], prophylaxis for COVID-19 [91 (29.9%)] and fear of infection at the hospital, [83 (27.3%)] as shown in Fig 1.

### Relationship between demographic characteristics and covid-19 related associated factors for ASM

The risk factor analysis showed that the socio-demographic characteristics of participants that significantly correlated with COVID-19 related risk factor for ASM were age and occupation (p<0.05) as indicated in Table 4.

**Table 4:** Relationship between demographic characteristics (ASM) and COVID-19 related associated factors for ASM (n = 400).

		COVID-19 Related Reasons for Antibiotic Self Medication				Chi-Square Test		
Variable	Category (n = 304)	Fear of Infection at the Hospital	Prophylaxis for COVID-19	Exposure to COVID-19 Patient	Symptoms of COVID-19	Chi-square Value	df	p-value
**Gender**	Male (n = 125)	29.6%	27.2%	10.4%	32.8%	2.35	3	0.503
	Female (n = 179)	25.7%	31.8%	6.7%	35.8%			
**Age**	18–24 (n = 84)	16.7%	34.5%	8.3%	40.5%	44.60	15	0.001
	25–35 (n = 86)	17.4%	32.6%	8.1%	41.9%			
	36–45 (n = 63)	30.2%	30.2%	4.8%	34.9%			
	46–55 (n = 51)	58.8%	21.6%	9.8%	9.8%			
	56–65 (n = 12)	16.7%	33.3%	16.7%	33.3%			
	> 65 (n = 8)	37.5%	0.0%	12.5%	50.0%			
**Marital Status**	Single (n = 118)	21.2%	31.4%	7.6%	39.8%	12.24	6	0.057
	Married (n = 175)	29.1%	29.1%	8.6%	33.1%			
	Divorced (n = 11)	63.6%	27.3%	9.1%	0.0%			
**Education**	Illiterate (n = 25)	16.0%	40.0%	0.0%	44.0%	9.41	9	0.401
	Basic (n = 123)	30.9%	28.5%	8.1%	32.5%			
	Secondary (n = 119)	27.7%	28.6%	7.6%	36.1%			
	Tertiary (n = 37)	21.6%	32.4%	16.2%	29.7%			
**Occupation**	Public Sector (n = 84)	28.6%	27.4%	10.7%	33.3%	22.92	12	0.028
	Private Sector (n = 90)	21.1%	30.0%	5.6%	43.3%			
	Self Employed (n = 82)	39.0%	30.5%	9.8%	20.7%			
	Unemployed (n = 29)	24.1%	37.9%	6.9%	31.0%			
	Student (n = 19)	5.3%	26.3%	5.3%	63.2%			
**NHIS**	No (n = 76)	19.7%	36.8%	7.9%	35.5%	3.78	3	0.286
	Yes (n = 228)	29.8%	27.6%	8.3%	34.2%			

### Knowledge, attitude and course of treatment with antibiotics

**Fig 2:**
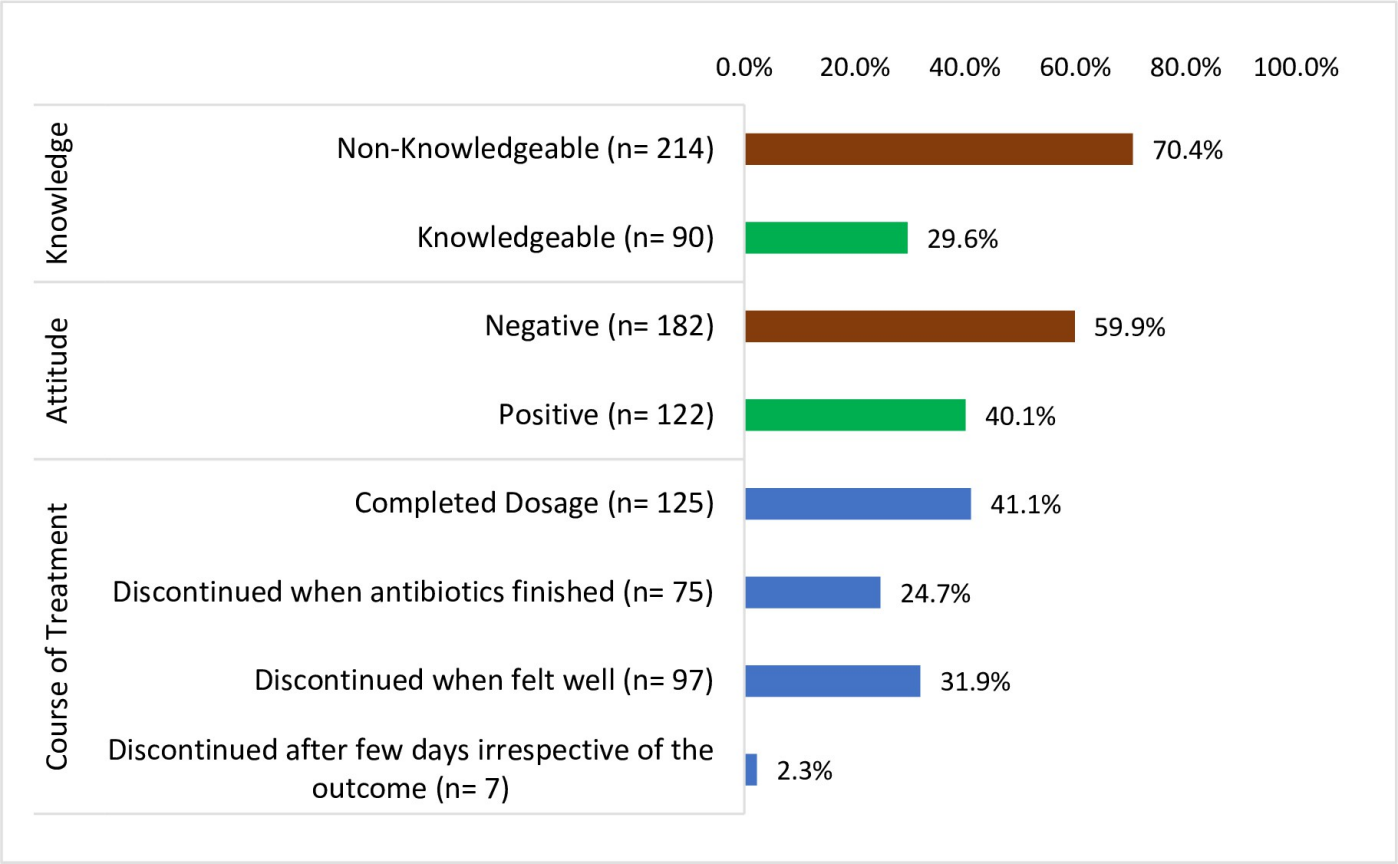
Knowledge, attitude, and course of treatment with antibiotics (n = 304).

Two hundred and fourteen (214), 70.4% of participants were ignorant about antibiotics and the negative consequences of antibiotic self-medication while [182 (59.9%)] had a negative attitude towards antibiotic usage. Majority [125 (41.1%)] completed dosage and [97 (31.9%)] discontinued the treatment when felt well as illustrated in Fig 2.

### Condition of health that necessitated antibiotic self-medication

**Fig 3:**
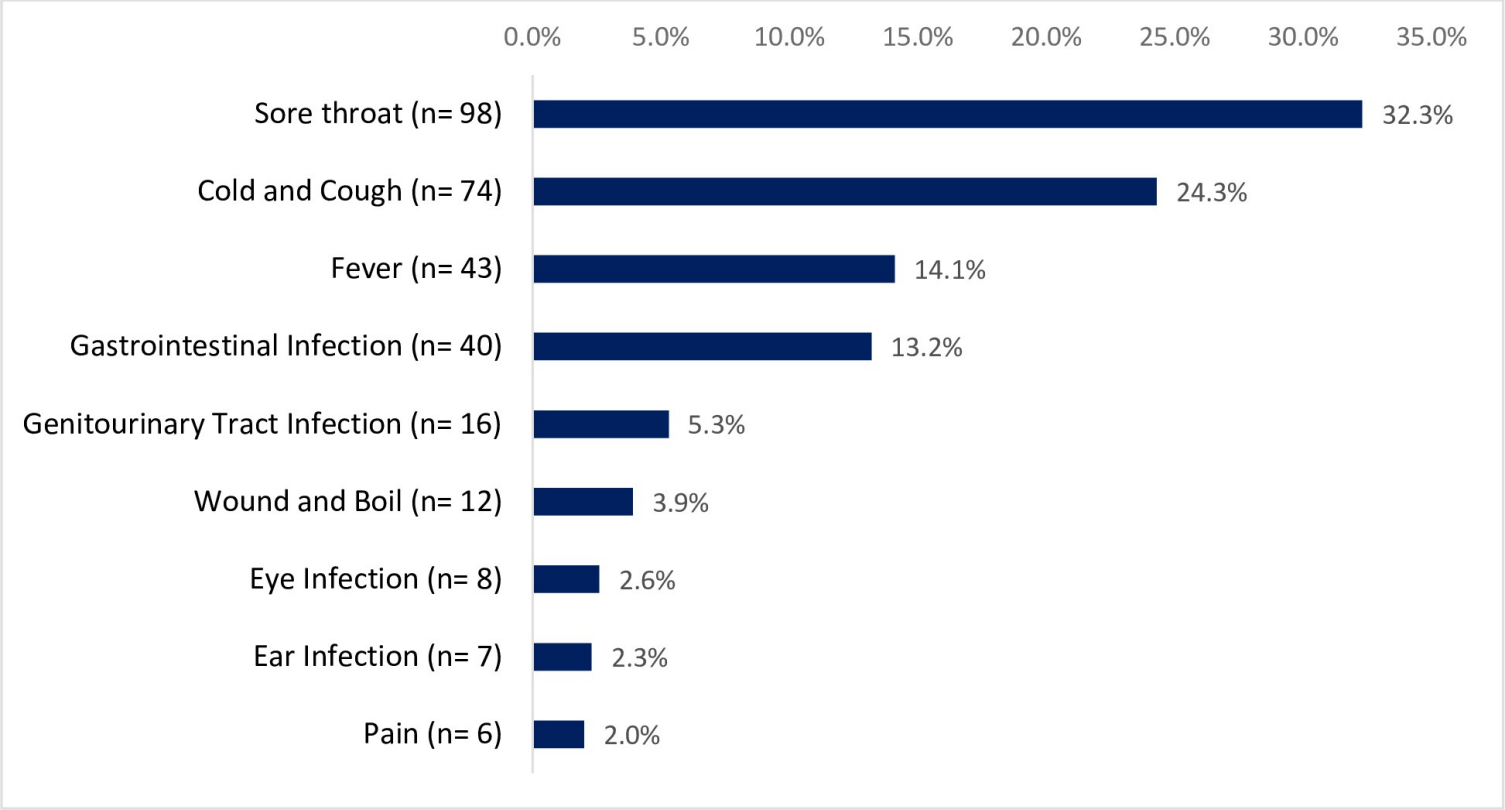
Condition of health that necessitated antibiotic self-medication (n = 304).

Antibiotic self-medication was used for sore throats [98 (32.3%)], cold and cough [74 (24.3%)], fever [43 (14.1%)], gastrointestinal infection [40 (13.2%)], genitourinary tract problems [16 (5.3%)], wound and boil [12 (3.9%)], eye infection [8 (2.6%)], ear infection [7 (2.3%)] and pain 6 (2.0%) as shown in Fig 3. A majority of [172 (57.6%)] of the antibiotic self -medication was administered due to respiratory tract symptoms such as sore throats, cold and cough.

### Sources of antibiotics and information about antibiotics

**Fig 4:**
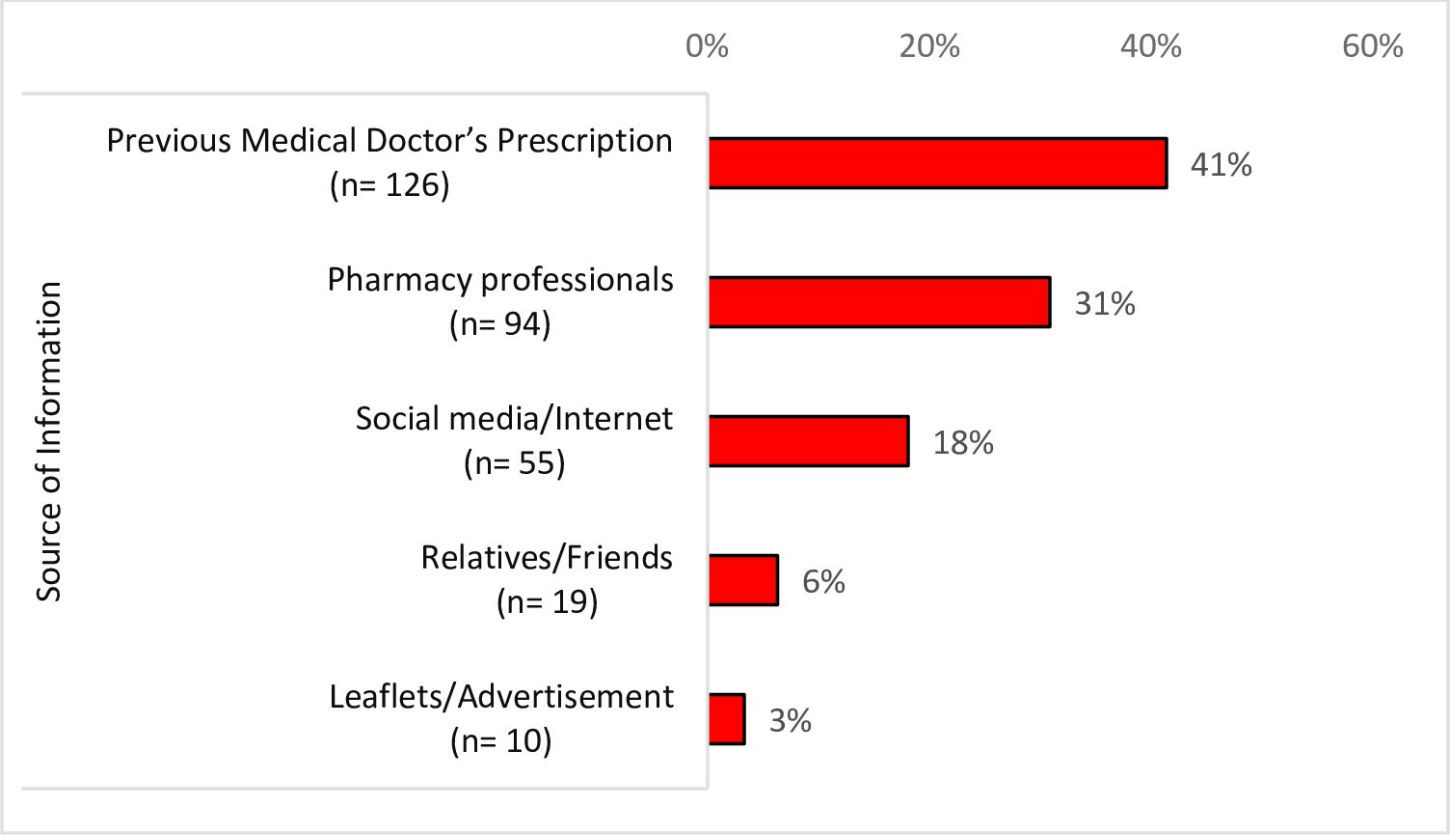
Source of information about antibiotics (n = 304).

The antibiotics used by the respondents for self-medication were mostly obtained mostly from pharmacy shop, [242 (79.6%)] as shown in Table 5. The source of information for majority of the respondents who practiced antibiotic self-medication was previous prescriptions, [126 (41%)] as indicated in Fig 4.

**Table 5:** Source of antibiotics (n = 304).

Source of Antibiotics	Count	Percent
Pharmacy Shop	242	79.6%
Relatives/ Friends	19	6.3%
Leftovers from previous prescriptions	43	14.1%

### Commonly used antibiotics for self-medication

**Fig 5:**
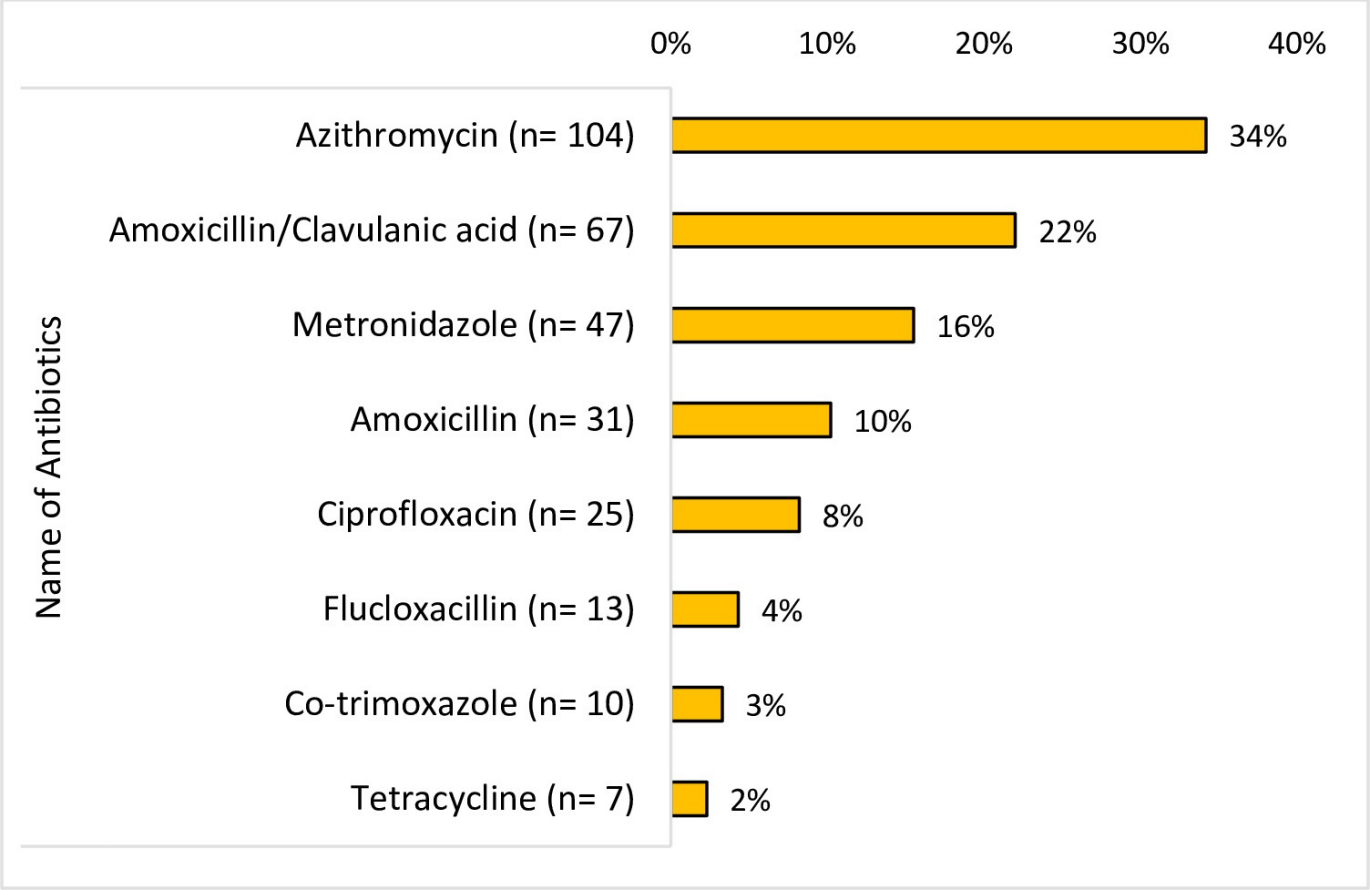
Commonly used antibiotics for self-medication (n = 304).

A majority of those who practiced ASM, 104 (34%) used azithromycin [104 (34%)], followed by 67 (22%) amoxicillin/clavulanic acid [67 (22%)], with [7 (2%)] using tetracycline as indicated in Fig 5.

## Discussion

The current study focused on the prevalence and associated factors of antibiotic self-medication (ASM) among adult residents during the COVID-19 pandemic in Tema, Ghana. Before the COVID-19 pandemic, antibiotic self-medication was a well-known public health threat and a common practice in Ghana with relatively high prevalence rate as reported by Kretchy *et al*., 2021 [4]. The study revealed a high prevalence of ASM among residents of Tema, Ghana, during the COVID-19 pandemic, with 76% of respondents admitting to practicing ASM within the previous 12 months. This prevalence underscores the urgent need for public health interventions aimed at curbing this behavior. This outcome was comparable with the studies of Khan *et al*., (2021) on antimicrobial consumption among patients with COVID-19 [14]. It was also consistent with the ASM prevalence range of 24% to 79% in Nigeria and other low- and medium-income countries (LMICs) in Africa [15].

The current prevalence rate was higher than the prevalence of self-treatment with antibiotics ranging from 20.8% to 45.8% in Eastern Mediterranean Region (EMR) countries during the COVID-19 pandemic [16]. The differences in prevalence may be due to differences in antibiotic awareness in the population or study design.

The study identified several socio-demographic factors significantly associated with ASM, including gender, age, marital status, education, occupation, and National Health Insurance Scheme (NHIS) subscription.

This is consistent with the previous studies in Ghana [4, 17] and some EMR countries [16].

Residents in Tema between the ages of 18–25 practiced ASM more than other age group. Males and singles turn to self-treat with antibiotics than females and married residents respectively. This may be because male working class abhor wasting time in long queues in the hospital. It seemed residents with tertiary education had a lower likelihood of participating in ASM, according to our study. This may imply residents with tertiary education may comprehend the impact of ASM on the emergence of antibiotic resistance and its associated health and economic importance.

Ironically other studies have established that students pursuing health related programmes in tertiary institutions tend to have a higher prevalence in ASM than the public [18–21]. The better appreciation of diseases and treatment options might have influenced their decisions not to consult physicians for treatment.

The results of this study indicated that residents who were self-employed or in the private sector tend to practice ASM during the COVID-19 pandemic. The unemployed and students also had the propensity of antibiotic self-treatment. The apparent high cost of hospital care is likely to influence the low- and irregular-income earners to opt for ASM instead of prescription-based treatment.

Prior to the COVID-19 pandemic, it was expected that residents with NHIS subscription will visit the hospital for medication as reported in Ghana [4], Ivory Coast [22] and India [23]. However, the current study indicated that residents with NHIS practiced ASM more than those without NHIS subscription during the COVID-19 pandemic. This may be attributed to the inconveniences and fear of contracting COVID-19 infections in the hospital.

The reasons for the high prevalence of ASM could be COVID-19 related as well as other medical and non-medical related factors. The study has established that, most residents in Tema practiced ASM because of treating symptoms of COVID-19 (34.5%), as a prophylaxis for COVID-19 (29.9%), fear of infection at the hospital (27.3%) and exposure to COVID-19 (8.2%).

Aside the COVID-19 related reasons, other medical related reasons for ASM included previous successful experience (44.1%), easy access to medication (30.3%), minor sickness (15.5) and emergency use (10.2%). These medical related reasons for ASM during the COVID-19 pandemic is consistent with the situation prior to the COVID-19 disease in Ghana [4] and Eritrea [24].

The other non-medical related reasons why residents practiced ASM during the COVID-19 pandemic included convenience (46.4%), long queues at the hospital (28.9%), cost saving (23.4%) and no access to medical care (4.0%). This is consistent with previous studies in Ghana [4] and other EMR countries [16].

About 70% of participants were ignorant about antibiotics and the negative consequences of antibiotic self-medication while 182 (59.9%) had a negative attitude towards antibiotic usage. This observation was consistent with a study in Eritrea where 84% of the participants had inadequate knowledge of antibiotics though majority had understanding that antibiotics are indicated for bacterial infections and not for viral infections [24]. The negative attitudes of not completing dosage and discontinuing treatment when felt well indicated in the current study was consistent with most countries in Southeast Asia (SEA) [19]. Policy makers and civil societies must emphasis health promotion targeted at behavioral change with regards to the rightful use of antibiotics to prevent multi-drug resistance and other public health issues associated with ASM.

The most often reported health conditions that required ASM in the current study region were respiratory tract infections such as sore throat, cold and cough. This was in line with the findings of previous study in other countries [16]. Others health concerns that necessitated ASM included fever and gastrointestinal tract infections, such as diarrhea, vomiting, and nausea was comparable to previous study in Ghana [4]. These infectious diseases are often related poor environmental conditions and poor air quality. Often, people who experience these symptoms end up abusing antibiotics. It is recommended that targeted interventions, such as strict law enforcement and ongoing public education on the responsible use of antibiotics, be implemented to decrease the sale of these drugs without a prescription and from questionable sources.

Contrary to the established regulations, antibiotics are typically offered over the counter in Ghana and other LMICs in pharmacy shops by both professional pharmacists and untrained chemical dealers, as well as through peddling, making it quite simple to buy them without a prescription. A good number of respondents also use leftover antibiotics from previous usage. Many culprits of the antibiotic self-medication obtain their information from previous prescriptions, pharmacy shops and social media. This accessibility to antibiotic purchases by locals may be related to the low enforcement of antibiotic purchase laws that are prevalent in many sub-Saharan African, EMR and SEA countries [16, 19, 25, 26].

During the pre-COVID-19 era, penicillins (amoxicillin), cepaholosporins and fluroquinolones (ciprofloxacin) seemed to be the most prevalent self-medicated antibiotics in Ghana [4, 13] and other parts of the world [14, 16]. According to the current study, azithromycin (34%) and amoxicillin/clavulanic acid (22%) were the two most frequently used antibiotics for self-medication, with co-trioxazole (3%) and tetracycline (2%) being the least common. This was comparable to studies in Eastern Mediterranean Region and other lower-middle-income countries [16]. In Nigeria, however, amoxicillin and ciprofloxacin were the most used antibiotics for the management of COVID-19 [25]. Interestingly, Togo also reported low (1.2%) prevalence use of azithromycin in the treatment of COVID-19 [26]. This may be due to its relatively high cost in Togo [26].

Although antibiotics are not used to treat viral infections, the misuse of azithromycin in the treatment of COVID-19 may have stemmed from lack of proper treatment guidelines and misinformation from social media [27]. Other reasons may be due to the fear of secondary co-infection in the event of COVID-19 [28].

One critical implication of these findings is the need for targeted public health campaigns that address the specific motivations behind ASM. For instance, the study found that convenience and long queues at hospitals were major non-medical reasons for ASM, while fear of infection at healthcare facilities and the desire to treat COVID-19 symptoms or use antibiotics prophylactically were significant pandemic-related factors. Public health messages should therefore emphasize the dangers of ASM, particularly in the context of viral infections like COVID-19 and promote the proper use of healthcare services.

Additionally, easy access to antibiotics without prescriptions highlights a regulatory failure that needs immediate attention. Strengthening the enforcement of existing laws on antibiotic sales and improving the training of pharmaceutical staff could help reduce the availability of antibiotics for self-medication.

One of the main strengths of this study is its use of a random sampling technique and a researcher-assisted questionnaire, which helped to ensure a representative sample and reliable data collection. The study’s focus on a specific, well-defined population (adult residents of Tema) during a particular period (peak of the COVID-19 pandemic) provides a clear snapshot of ASM behaviors and associated factors during a critical time.

The comprehensive nature of the questionnaire, which included both socio-demographic information and detailed questions about ASM practices, is another strength. This allowed for a thorough analysis of the factors associated with ASM and provided valuable insights into the reasons behind this behavior.

Despite its strengths, the study has some limitations. Firstly, the cross-sectional design of the study means that it can only provide a snapshot of ASM practices at one point in time, without establishing causality. Longitudinal studies would be needed to determine whether the identified factors directly cause changes in ASM behavior over time. Secondly, the study relies on self-reported data, which is subject to recall bias and social desirability bias. Participants might not accurately remember or may underreport their ASM practices, leading to potential underestimation of the true prevalence of ASM.

Additionally, while the study included a large sample size, it did not achieve the initially proposed sample size of 499, which may limit the generalizability of the findings. The reduced sample size was due to the availability of respondents that met the inclusion criteria, which could introduce selection bias.

Future research should aim to address the limitations of this study by employing longitudinal designs to track ASM behaviors over time and establish causal relationships. Studies should also consider using more objective measures of ASM, such as pharmacy records, to complement self-reported data and reduce bias.

Moreover, research should explore the impact of specific interventions aimed at reducing ASM. For example, studies could evaluate the effectiveness of public health campaigns, changes in antibiotic regulations, and improvements in healthcare access on reducing the prevalence of ASM.

Finally, expanding the scope of research to include other regions would help to understand the broader patterns of ASM and identify common factors and effective interventions across different contexts. Comparative studies between urban and rural areas, or between countries with different healthcare systems and regulatory environments, could provide valuable insights into the global challenge of ASM.

## Conclusions

The study highlights a significant public health issue with ASM in Tema, Ghana, during the COVID-19 pandemic. The high prevalence of antibiotics self-medication among the residents in Tema underscore the need for targeted public health interventions, regulatory enforcement, and further research to address this behavior. Easy access to antibiotics without prescriptions highlights the need for regulation enforcement. The non-medical and medical factors of convenience, avoiding long hospital queues, previous successful experience, easy access to antibiotics, treating symptoms, prophylaxis, and fear of hospital infection motivating antibiotic self-medication practices require the implementation of behinmore effective strategies to combat antibiotic resistance and improve public health outcomes.

## Supporting information

S1 Checklist *PLOS ONE* clinical studies checklist. (DOCX)

S2 Checklist STROBE statement—checklist of items that should be included in reports of observational studies. (DOCX)

S1 File (DOCX)

S2 File (XLSX)
