# Supplementary material for: Self-medication with antibiotics during the COVID-19 pandemic: A cross-sectional study among adults in Tema, Ghana
Source: PLoS One. 2024 Jun 25;19(6):e0305602. doi: 10.1371/journal.pone.0305602 (PMC11198810; doi:10.1371/journal.pone.0305602)
Supplement: S1 File — (DOCX) [file pone.0305602.s003.docx]

**Questionnaire**

**Informed Consent**

This cross-sectional survey was aimed at determining the prevalence of antibiotic self-medication and the possible associated risks during the peak of the COVID-19 pandemic among adult residents of Tema in Ghana. Your honest responses will help in achieving the goals to enhance the rational use of antibiotics. Your name is not needed because the data will be treated as confidential.

**Written Informed Consent Section** - *Please read carefully*

I have decided to participate in this research on my own volition without any coercion.

**Signature:** ___________

Thank you for your willingness to participate in this study.

**Section A: Socio-demographic Characteristics**

**Instruction:** Circle or write the answer that corresponds with the best answer.

| S/N | Socio-demographic characteristics | Response |
| --- | --- | --- |
| 1 | Sex | A. Male |
|  |  | B. Female |
| 2 | Age | . |
| 3 | Marital status | A. Single |
|  |  | B. Varied |
|  |  | C. Divorced |
| 4 | Education | 1. Illiterate |
|  |  | 1. Basic |
|  |  | 1. Secondary |
|  |  | 1. Tertiary |
| 5 | Occupation | 1. Public Sector |
|  |  | 1. Private Sector |
|  |  | 1. Self employed |
|  |  | 1. Unemployed |
|  |  | 1. Student |
| 6 | NHIS subscription | 1. Yes . |
|  |  | 1. No |

**Section B: Questionnaire on Antibiotic self-medication during the COVID-19 pandemic.**

**Instruction:** Circle or write the answer that corresponds with the best answer.

| S/N | Statement | Response |
| --- | --- | --- |
| 1 | Have you ever taken antibiotics? | A. Male |
|  |  | B. Female |
| 2 | Have you ever treated yourself (self-medicated) with antibiotics? | A. Yes |
|  |  | B. No |
| 3 | How many times did you treat yourself with antibiotics in the past one year? | ………………………… |
| 4 | What was your medical related reason of self-medication with antibiotics? | 1. Previous successful experience |
|  |  | 1. Easy access to medication |
|  |  | 1. Emergency use |
|  |  | 1. Minor sickness |
| 5 | What was your non-medical related reason of self-medication with antibiotics? | 1. Long queues at the hospital |
|  |  | 1. Cost saving |
|  |  | 1. Convenience |
|  |  | 1. No access to medical care |
| 6 | What was your COVID-19 related reason of self-medication with antibiotics? | 1. Fear on infection at the hospital |
|  |  | 1. Prophylaxis for COVID-19 |
|  |  | 1. Exposure to COVID-19 patient |
|  |  | 1. Symptoms of COVID-19 |
| 7 | For which of the following complaint(s) did you use antibiotics? | A. Cold and Cough. |
|  |  | B. Sore throat |
|  |  | C. Fever. |
|  |  | D. pains |
|  |  | E. Genitourinary tract infection |
|  |  | F. Gastrointestinal infection |
|  |  | G. Wound and boil. |
|  |  | H. Ear infection |
|  |  | I. Eye infection |
| 8 | Where did you get or buy your antibiotics from? | 1. Pharmacy shop 2. Relatives/ Friends 3. Leftovers from previous prescriptions |
| 9 | Your source of information which guided your selection of antibiotics was based on | 1. Pharmacy professional |
|  |  | 1. Relatives/ Friends |
|  |  | 1. Leaflet / Advertisement |
|  |  | 1. Social media /Internet |
|  |  | 1. Previous medical doctor’s prescription |
| 10 | How did you know the dosage of antibiotics? | 1. By checking the package insert |
|  |  | 1. By consulting a doctor |
|  |  | 1. By consulting a pharmacist |
|  |  | 1. By consulting family members/friends |
|  |  | 1. From the newspapers, magazines, books, or TV programs |
|  |  | F. From the Internet |
|  |  | G. From my previous experience |
|  |  | H. By guessing the dosage by myself |
|  |  | I. Others (specify) |
| 11 | Did you ever change the dosage of antibiotics deliberately during the course of self-treatment? | A. Yes, always |
|  |  | B. Yes, sometimes |
|  |  | C. Never |
| 12 | When did you normally discontinue taking antibiotics? | A. Discontinued when felt well |
|  |  | B. Completed dosage |
|  |  | C. Discontinued after few days irrespective of the outcome |
|  |  | D. Discontinued when antibiotics finished |
| 13 | Please indicate the names of antibiotics you have ever taken for self-medication. | 1. Amoxicillin |
|  |  | 1. Ciprofloxacin |
|  |  | 1. Metronidazole |
|  |  | 1. Azithromycin |
|  |  | 1. Amoxicillin / Clavulanic acid |
|  |  | 1. Tetracycline |
|  |  | 1. Flucloxacillin |
|  |  | 1. Co-trimoxazole |
|  |  | 1. Others (specify): |
| 14 | What do you think about self-medication with antibiotics for self-health care? | A. Good practice |
|  |  | B. Acceptable practice |
|  |  | C. Not acceptable practice |
| 15 | Do you know what antibiotics are? | A. Yes |
|  |  | B. No |
| 16 | What are antibiotics used for? | A. Virus infection |
|  |  | B. Bacterial infection |
|  |  | C. Others (specify) |
| 17 | Are antibiotics good for common cold? | 1. Yes |
|  |  | 1. No |
